# Involving systems thinking and implementation science in pharmacists’ emerging role to facilitate the safe and appropriate use of traditional and complementary medicines

**DOI:** 10.1186/s12960-020-00493-9

**Published:** 2020-08-03

**Authors:** Joanna E. Harnett, Shane P. Desselle, Hao Hu, Carolina Oi Lam Ung

**Affiliations:** 1 The University of Sydney Pharmacy School, Faculty of Medicine and Health, The University of Sydney, Sydney, New South Wales Australia; 2 College of Pharmacy, Touro University California, 1310 Club Drive, Vallejo, CA 94592 USA; 3 State Key Laboratory of Quality Research in Chinese Medicine, Institute of Chinese Medical Sciences, University of Macau, Macao SAR, China

**Keywords:** Traditional medicines, Complementary medicines, Pharmacist, Systems thinking, Implementation science

## Abstract

The use of traditional and complementary medicines (TM/CMs) has become an increasingly popular part of healthcare and self-care practices across the world. While the benefits and risks of many TM/CMs are yet to be fully evaluated, their prevalent use without consistent oversight has not been fully addressed by the public health sector. Pharmacists play an integral role in contributing to public health. Discussion about integrating TM/CMs into the professional practice of the pharmacist began over two decades ago. Nevertheless, TM/CMs are predominantly managed as “retail products” and are not integrated into pharmaceutical care and practice. While some isolated measures towards integration have been proposed, there remains no consensus on how to deliver pharmaceutical care in a coordinated, systematic manner. Systems thinking approaches are needed to formulate and implement strategies that change pharmacists’ practice related to TM/CMs. Such approaches will ultimately reduce risk, optimize patient care, and result in better health outcomes.

## Background

The World Health Organization (WHO) defines complementary medicine (CM) as a broad set of healthcare practices that are not part of that country’s own traditional or conventional medicine and are not fully integrated into the dominant healthcare system. The term is often used interchangeably with traditional medicine (TM) [1]. TM is described as having a long history of use and *‘the sum total of the knowledge, skill and practices based on the theories, beliefs and experiences indigenous to different cultures, whether explicable or not, used in the maintenance of health as well as in the prevention, diagnosis, improvement or treatment of physical and mental illness; and T&CM merges the terms TM and CM, encompassing products, practices, and practitioners.'* For the purposes of this commentary, and within the context of pharmacy, the term TM/CMs will refer to products that are used for medicinal purposes including herbal, nutritional, vitamin and mineral supplements, and homeopathic medicines.

TM/CMs are an increasingly prevalent component of healthcare and self-care practices across the world [1]. People choose TM/CMs for health maintenance as well as disease prevention and treatment, with varying prevalence rates across regions. For many people who live in less developed countries, traditional medicines are sometimes the only source of healthcare. As new evidence emerges regarding the safety and effectiveness of TM/CMs, their increasing contributions to patient health outcomes in different regions of the world will become apparent.

The safe use of TM/CMs is an ongoing debate associated mainly with concerns about the quality of the products and potential adverse events. For people living with chronic or other health issues, delaying known effective treatments and the risk of experiencing adverse drug reactions and drug-herb interactions may be increased [2]. Some of these risks may be confounded by the emergence of integrative medicine that is practiced in different ways across cultures and health systems. The term “integrative medicine” has been included in the 2019 WHO TM/CM report to cover integrative approaches that involve using both T&CM and conventional medicine [1]. Currently, a separate WHO project is underway to define and understand integration as well as integrative medicine, with a view to guiding countries on best practices for integrating T&CM (including TM/CMs) into national health systems [1]. Collectively, there is a public health need to harness the potential benefits and minimize risk associated with TM/CM use around the world [3].

The pharmacist’s role in contributing to public health by ensuring the safe and appropriate use of medicines is well established [4]. Discussion regarding broadening the scope of pharmacists’ professional practice to include TM/CMs began over two decades ago [5]. International and national professional pharmacist organizations also advocate the inclusion of TM/CMs into pharmacists’ scope of practice. In general, pharmacists recognize the relevance of TM/CMs to their daily practice, the needs of consumers, and are keen on stepping up their professional role in the delivery of more responsible pharmaceutical services [6–8].

Nevertheless, pharmacists still do not routinely initiate conversation with consumers about their use of TM/CMs [9–11]. No consensus has been reached about the practicalities and processes required to engage pharmacists in caring for people who use TM/CMs. A substantial gap remains between the responsibilities that have been proposed in relation to TM/CMs, and what actually takes place in day-to-day pharmacy practice. With a view to progressing this important area, research was recently undertaken in the Unites States of America [7, 10], Australia [6, 11], and China [8, 9], with each study representing different manifestations of integrative medicine within the respective countries pharmacy systems and culture context. Drawing on these findings, this commentary aims to posit reasons why pharmacists have not fully engaged in adopting a public health role in relation to TM/CMs, and calls for a coordinated scientific and systematic approach to the implementation of this important professional role.

## Barriers faced by pharmacists are multifaceted but coherent

Employing a grounded therapy methodology, the Australian key stakeholder study identified multiple barriers impeding optimal pharmacist care, including insufficient knowledge about TM/CMs, reactive (rather than proactive) attitude towards TM/CM inquiries, a lack of access to reliable information sources, insufficient skills to interpret the available evidence, poorly defined role, and poor inter-professional communication with prescribers [6]. Similarly, pharmacists in the USA complained about ambiguous expectations and deliverables in relation to TM/CMs. They purported that proposed responsibilities by professional bodies were idealistic and impractical to execute without appropriate support in TM/CM education and training, defining clear roles and responsibilities, and access to evidence-based information [11].

Pharmacists in China were particularly concerned about lack of knowledge at the interface between TM/CMs and conventional medicines given the high degree of integrative medicine in that region [8]. They further questioned the motivation of pharmacists to take on additional responsibilities in the absence of reasonable remuneration for these added roles. Moreover, the regulatory framework in China that explicitly separates the scope of practice for the two pharmacist workforce cadres (one in conventional medicine and the other in traditional Chinese medicine) acts as a deterrent to pharmacists in taking on additional professional duties. These fundamental barriers to pharmacists engaging full professional duties related to TM/CMs were not isolated issues and inextricably associated with multiple factors controlled by related sectors. Therefore, to progress this important public health area, a scientific approach to implementing a coordinated and strategic development is critical.

## Importance of a systems thinking approach

Addressing individuals’ and patients’ needs is part of a complex system involving not just pharmacists but also other healthcare professionals, the organizations that employ them, and the larger environment of laws, rules, remuneration, accreditation, and professional training institutions [12]. However, in practice, decisions regarding interventions often lack systematic planning and review of the best evidence to identify effective approaches. Barriers to implementing new or extended pharmaceutical services include the political environment, deficits in timely research, information systems, resources, leadership, and the required competencies. This involves bringing together interdependent smaller systems to work in the same process towards common goals.

To move this facet of practice forward, the isolated actions currently adopted by various stakeholders should be transposed into a coordinated and collaborative “systems thinking” approach. For this, systems thinking is a tool that allows key stakeholders to map the health system, identify where some of the key impediments and challenges lie, and design synergistic and system-ready interventions. This approach advocates for transdisciplinary, translational, and network-centric approaches needed to improve the understanding of and address the challenges among the complex inter-relationships among variables. The use of systems thinking approaches encourages relationship-building across various disciplines so as to achieve a common set of relevant goals and objectives on public health matters [13]. An example of such approaches is the strategic model developed for integrating TM/CMs into professional pharmacy practice in Australia [11]. This model outlined key stakeholders and their relationships, then proposes the reforms and actions that are required to realize progress in this important area which will be explained further later in this paper. High-level engagement and sufficient resources to sustain and monitor interventions were highlighted, and key stakeholder partnerships were well positioned to enact and enable all the necessary actions.

## Next step: facilitating systematic changes using implementation science

Another major challenge here is to implement strategies that translate much-needed services into sustained routine practice. This is exemplified by professional organizations publishing a set of responsibilities for pharmacists that are essentially a standpoint rather than a practical recommendation for systematic approaches to facilitate high-quality implementation. There needs to be a scientific approach to identify the range of factors that are likely to facilitate administration of an intervention. More importantly, determining the indicators and methods to measure changes and demonstrate any clinical, humanistic, and economic outcomes associated with pharmacists’ new practice is essential to support the sustainable development of the intervention or service. Implementation knowledge and strategies must be incorporated to promote intervention validity, while collecting the data necessary for establishing evidence-based practices to optimize such intervention in patient care delivery [14]. For this, the discipline of implementation science, which has already been used frequently as a primer to guide the successful development of professional pharmacy services into routine practice, should also be used to address the research-to-practice gap and accelerate pharmacists’ practice change related to TM/CMs.

**Fig. 1:**
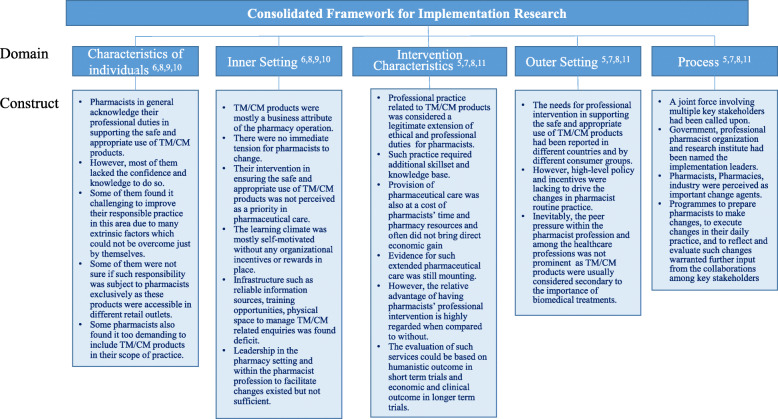
The Consolidated Framework for Implementation Research about pharmacists’ emerging role to facilitate the safe and appropriate use of TM/CMs

Implementation science is comprised of two major components: implementation practice and implementation research. With the practice of implementation, the prime question here is “how to implement.” To put the “how” factors into perspective, consider the Consolidated Framework for Implementation Research (CFIR) [15], one of the implementation science theoretical approaches often used to guide service implementation in pharmacy, to describe the structured understanding of such findings derived from studies the USA, Australia, and China (Fig. 1). Implementation science serves a foundation for a systems thinking approach in knowing that practice change in one area will impact practice in other areas. For example, how pharmacists will gather information on TM/CMs from consumers will subsequently impact how they counsel patients upon dispensing a conventional product, and these two activities are treated as part of a continuum of care, rather than as discrete, unrelated functions. In fact, much of the current research on this topic has investigated the “how” questions in various contexts.

For future research, implementation research that focuses on evaluating the most effective approaches for implementing an intervention is paramount. Implementation research can be employed at two levels: (1) evaluate approaches to adopt interventions that aim to change the quality of pharmacists’ practice in relation to TM/CMs and (2) evaluate pharmacist’s interventions that aim to improve patient outcomes and benefit population health through safeguard the safe and appropriate use of TM/CMs. For instance, the current understanding about the knowledge-practice gaps can be used to formulate a systematic strategy appropriate for a specific context, which may be a combination of developing practice standards, training design, competence accreditation mechanism, and/or reasonable remuneration system. The questions yet to be answered shall focus on “what” strategies will be most effective in optimizing pharmacists’ performance and thus healthcare outcomes, and “how” to sustain and scale up services that have been found to be effective. Future research design on implementation strategies and intervention effectiveness will benefit by adopting the five implementation domains: an exploration phase, a preparation phase, a testing phase, a full implementation phase, and a sustainability phase [16]. A better understanding and connection of the research designs of each stage will help improve the adoption, appropriate adaptation, delivery, and sustainment of any interventions proposed to facilitate advances in pharmacists’ professional practice related to TM/CMs and to improve health outcome at patient and population level.

## How to apply systems thinking and implementation science

A systems thinking approach in the form of strategic models has been developed following studies conducted in the USA, Australia, and China. These models have been proposed for use in order to address the issues identified at the interface between TM/CMs and the pharmacist professional practice [7, 8, 11]. The elements within each of these models differed according to the local context. However, each model proposed an interdisciplinary approach among a range of stakeholders in order to implement effective change. The strategic model proposed for implementation in Australia consisted of developments required in education and training, building the TM/CM evidence base, the development of reliable and accessible TM/CM information resources, and reinforcing workplace support. For this to be implemented, stakeholders from professional pharmacy organizations, universities, government, pharmacy owners, and pharmacists would take on relevant elements within the strategy to address.

According to the Australian model [11], with regard to implementing education and training, the first step was for the professional pharmacy organizations (PPO) to take the lead and make recommendations about the TM/CM practice standards. The practice standards, however, should be the outcome of a coordinated effort among stakeholders in providing the resources needed. In particular, Australian pharmacists clearly valued the quality of non-biased training provided by the universities. PPO would need to communicate with pharmacy schools (PS) about the recommended practice standards. The PS were then expected to design and incorporate TM/CM teaching in the syllabus to equip pharmacy graduates with CM competency that aligned with the practice standards. Such relational dependent outcomes reaffirm that any developments by individual stakeholders are unlikely to be successfully implemented if they are individual projects conducted in isolation.

While a systematic approach to developing the aforementioned strategy is a critical phase to informing and implementing changes in pharmacists’ practice, it is important to consider the indicators and methods that will be required to measure the impact of such change. These will be essential to informing and supporting sustainable developments in this important area. Therefore, a well-defined list of performance indicators to reflect both output and outcome of any changes implemented is critical and considered an important next step in this body of research.

To do this, a staged approach involving a small-scale project in the form of a controlled study is recommended, prior to implementation in a larger public setting. Simple measurements such as pharmacist’ uptake of training, improvement in the level of TM/CM knowledge, changes in their attitude, and even the quality and accuracy of pharmacist TM/CM practice pre- and post-training would provide some indications about the effectiveness of the training actions.

Central to the purpose of this work is people’s wellbeing and safety. Therefore, evaluating consumer’s experience and encounter with the pharmacist is critical to understanding the impact of implementing such change. Consumers’ level of satisfaction about the pharmacist TM/CM practice, clinical outcomes (e.g., the prevention or reduction of drug/herb adverse events), and/or economic outcome (e.g., cost-savings associated with improved polypharmacy behavior) should be measured to demonstrate the implications of training and the actual value of pharmacist’ TM/CM practice.

Collectively, we posit well-redesigned research methodology is essential to connect the various phases of exploration, preparation, testing, implementation, and sustainability of interventions in improving pharmacist practice related to TM/CMs.

## Conclusion

The pharmacist’s professional role in ensuring the safe and appropriate use of TM/CMs is central to public health. A systems thinking approach is needed to fill the current gaps acting as barriers and to formulate and prioritize actions involving key stakeholders at different levels and stages. Implementation science offers a solid framework to plan, execute, review, and evaluate the sustainable development of pharmacists’ roles to ensure the optimal use of TM/CMs. Irrespective of a country’s culture and regulatory framework, the unification of all stakeholders in the medication use process should make an indelible impact on public health.

## Data Availability

Not applicable

## Abbreviations

TM/CMs: Traditional and complementary medicines
CFIR: Consolidated Framework for Implementation Research
